# Medical Opinion and Movement

**Published:** 1910-10-29

**Authors:** 


### Medical Opinion and Movement.

### Anæsthesia for Gastric Crises of Tabes.

The effects obtained by spinal anæsthesia suggested to Dr. Sapatch-Sapotchinsky, of St. Petersburg, the idea of employing it in cases of gastric crises of tabes dorsalis. His first trial of the treatment was on a patient suffering from very intense crises accompanied by severe vomiting and insomnia and persisting generally for two or three weeks. One injection of 2 c.c. of the anæsthetic solution, after previous evacuation of an equal amount of cerebro-spinal fluid, not only relieved the immediate symptoms, but prevented their recurrence for three weeks. A second injection was made on the return of the symptoms with the same success, and since then there has been no recurrence, and the patient has been able to resume his occupation. In three other similar cases one injection has been sufficient to produce the same effects. In consequence of these results the author has since tried the treatment in other tabetics, in whom the crises were not so severe or lasting, but nevertheless exhausted the patients by their frequent repetition. In two such cases the injections have led to a diminution both in the intensity and frequency of the attacks. In two other cases, in which there was marked general debility, the improvement was only small and of short duration. But in a private patient, who suffered from severe gastric crises recurring five or six times in the course of a year and who, by recourse to morphia to relieve the condition, had become a morphomaniac, the spinal injections were so successful that he was induced to give up the morphia altogether.

### Danger of Menthol Preparations for Infants.

A useful note of warning has been raised by several physicians recently against the administration of menthol preparations, especially in the form of nasal instillations, to infants. Dr. P. F. Armand-Delille, of Paris, first drew attention to the fact that they are liable to produce a condition of severe asphyxia, and reported a case of an infant aged three months suffering from capillary bronchitis, for whom he prescribed, in addition to other therapeutic measures, nasal instillations of 1 per cent. menthol in oil. Fortunately he made the first application himself. It was immediately followed by a severe spasm of the glottis with apnœa, violent respiratory efforts, and cyanosis. These phenomena were accompanied by an excessive mucous secretion in the naso-pharynx, necessitating continual swabbing with cotton wool on the end of the finger. By this means, combined with a hot bath, the infant recovered in the course of a quarter of an hour. A similar case of violent spasms following a nasal instillation with menthol in an infant is recorded by Dr. Malebran, of Mentone. Two further cases of a like nature are reported by Dr. L. Mayet, of Lyons. Finally, Dr. W. Koch, of Fribourg, in Brisgau, relates a case of severe laryngeal spasm following the use of menthol. The patient was an infant aged three weeks, affected with an acute coryza. He prescribed the application of a menthol preparation to the nasal fossæ by means of cotton-wool swabs on the end of a match. Immediately on the first application the infant developed signs of intense asphyxia, with complete apnœa and cyanosis of the face, convulsions and foaming at the mouth. The condition was only relieved in the course of half an hour by artificial respiration, and by constantly freeing the mouth and pharynx of the excessive mucous secretion. Such examples are a sufficient warning against the use of menthol in infants or even children at all of tender years.

### A New Sign in Chest Affections.

At a meeting of the Académie de Médecine Dr. Hirtz drew attention to a new diagnostic sign for certain affections of the pleura or lungs, which is obtained by percussion of the chest in a state of sustained inspiration. With the patient breathing at first in a normal manner the chest is percussed, and any area of dulness or partial dulness or high-pitched note is observed. Then the patient is instructed to take a deep and sustained inspiration, and at the same time the chest is percussed again and any change in the area of dulness noted. If the dulness is due to a pulmonary congestion, œdema, or a pleuro-pneumonia, the dull area becomes more or less resonant on deep sustained inspiration, whereas if there is a pleural effusion there is no such marked change in the note. In this way, therefore, a differential diagnosis between some such pulmonary condition—congestion, œdema, or pleuro-pneumonia—and pleuritic effusion may be made without having recourse to an exploratory puncture. Similarly, it is possible to decide by this sign in the course of a pleurisy whether fluid is present or not. According to the author, this sign may enable one to differentiate certain conditions of congestion at the pulmonary apices in cases of cardiac or Bright's disease from tubercular lesions, with which they are liable to be confounded. Although the sign may prove of value to clinicians, it may be doubted whether it would always give such unequivocal results as the author hopes for.

### Exophthalmic Goitre.

Dr. Van Lier has carried out a series of researches on the blood of sixty-two patients suffering from Basedow's disease. Contrary to the observations of Kocher, Gordon and Jagic, and of Bühler, who describe a leucopenia, he is in accord with Dr. Caro in finding a normal or increased proportion of leucocytes (5,000 to 14,000). In all cases, both in the definite cases of the disease and in the abortive cases, there is an increase in the large mononuclears amounting to as much as 18 per cent. But whereas in the abortive forms of the disease the other varieties of leucocytes are pretty well in normal proportion, in the definite cases the lymphocytes almost invariably exceeded 30 per cent., and the polynuclear neutrophiles were considerably diminished, falling in some cases below 30 per cent. In ten patients affected with parenchymatous or colloid goître the blood count was normal. Three myxœdematous patients treated with thyroidine showed a lymphocytosis. After thyroidectomy, in cases not cured by operation, the same condition of the blood persists, but in cases that are completely cured the blood count is normal. Both patients in whom the author found the largest increase in lymphocytes and the smallest number of polynuclear neutrophiles died, one after thyroidectomy and the other after ligature of the superior thyroid artery. Both died of cardiac failure twenty-four hours after operation. Dr. Caro has made similar observations. Dr. Van Lier advises therefore against operation on patients in whom the number of lymphocytes amounts to 40 per cent. and that of the polynuclear neutrophiles falls to 45 per cent. or lower.

### Summer Diarrhœa.

In a paper recently read before the British Medical Association, Dr. J. H. Clements, of Bockonham, detailed the results of an investigation into forty-four cases of summer diarrhœa in a northern town in the year 1909. These cases were notified from forty-two houses, there being two sets of twins, and forty of them occurring between August 9 and September 9. In several houses adults or older children suffered from the disease, but were not included in the count, which only related to children under two years. The secondary cases were probably infected from a common source and not directly from the first case, for the same care was taken with the stools and linen as in the case of typhoid fever. In the great majority of the infected houses the yards were unpaved and the conveniences were privy middens. In the few cases in which the house was provided with a water-closet there were privy middens in the adjoining yards or close by. Speaking generally, the cases occurred in parts of the town where the housing was of the poorest type, where the sanitary arrangements were least satisfactory, and where there was evidence of neglect and want of cleanliness within and without the house. A rough estimate was made of the number of flies in the infected houses, and flies were collected from fifteen cases for bacteriological examination. In every house where diarrhœa occurred there were numbers of flies, and some of the houses were infested with them. The examinations proved that there could be little doubt that the fly reared in a manure heap had its alimentary canal well stocked with whatever organisms the manure might contain, and these organisms probably continued to multiply during the adult life of the fly, and got deposited wherever it chanced to alight. Dr. Clements expressed the view that this fact alone should suffice to cause the fly to be regarded with suspicion. During the period of fly prevalence some attempts were made to kill the flies in houses where they were the greatest nuisance. Sprays of various kinds were used, and formalin both in the form of vapour and spray was tried; the vapour did not kill the flies, and the spray was so unpleasant and irritating to the person using it that it had to be given up. A spray of izal proved to be more successful; it did not immediately kill the flies, but when sprayed over them they became stupefied and fell to the ground, where they could be swept or gathered up with a cloth and thrown into the fire. By the use of izal spray all the flies in several houses were killed.

### Minor Symptoms in Diabetes Mellitus.

Dr. Otto J. Stein, in the *Journal of the American Medical Association*, calls attention to a number of symptoms occurring in the nose, throat, and ear in connection with diabetes mellitus which are commonly overlooked. Thus, with reference to the teeth, an inflammatory condition around the roots, or delay in healing after extraction, may be noticed. This is due to the sugar in the blood tending to delay healing and to encourage pathological changes, rather than to acidity of the mouth secretions. Other oral symptoms comprise aphthous stomatitis, thrush, hyperæmia of the buccal mucous membrane, and a large reddish tongue covered with a black fur down the centre. In the throat, owing to the adherence of the secretions to the mucous membrane, a glazed appearance may be produced. Ulcers may occur on the tonsils, on the pillars of the fauces, and on the posterior pharyngeal wall, and are painful. They may be diagnosed from tuberculous lesions by the comparative readiness with which they heal. In the opinion of some authorities a dry pharyngitis, associated with languor, mental dulness, furunculosis, and cramps, is indicative of diabetes mellitus. In the nose the only characteristic symptom is a slightly cyanotic reddening resembling that met with in cases of heart disease associated with alcoholism. The author, however, believes that a number of other symptoms occur but have not yet been accurately described, such as hypersecretion, hyperæmia, itching, sneezing, and paræsthesiæ of the mucous membranes. In the ear, furunculosis of the external meatus is fairly common; cyanosis similar to that found in the nose is somewhat characteristic. Eczema, diffuse otitis externa, and suppuration are met with, but gangrene of the auricle is rare. A dusky purple bleb on the drum of the size of a millet-seed may result from a serous or hæmorrhagic effusion associated with disease of the kidney.

### Induction of Labour.

An interesting paper on this subject appears in the *Journal of Obstetrics and Gynæcology of the British Empire*, by Dr. Little, of Montreal. This author shares the prejudice which seems to exist everywhere except in Great Britain against the induction of premature labour for contracted pelvis; yet he recognises many more indications for the artificial production of labour than do, for example, most of the Continental obstetricians. He is strongly in favour of allowing most patients with contracted pelvis to go to term; he finds that in the last eighty-seven such women he has treated seventy had normal labours, and only seventeen required interference. If, after a trial of the second stage, the head fails to engage or is arrested, version, pubiotomy, or Cæsarean section are the alternatives. Much more often he would induce labour in a normal pelvis. The proportion of patients who go beyond term, and have dystocia from the unusual size of their babies, is, he thinks, larger than is generally supposed (it is given in Winckel's "System" as 16 per cent.), and for these he would induce labour at full term. Other indications are heart disease, eclampsia, placenta prævia, nephritis, chorea, tuberculosis, prolapse of cord, etc. The method used is one not described in English text-books. In the lithotomy position, and with full antiseptic precautions, the anterior lip of the cervix is fixed with a tenaculum. A medium-sized rectal tube, about 25 cm. long, is then inserted thus: Small holes about 0.3 cm. in diameter are made in the tube at intervals of 5 cm., and a flexible metal director is pushed into the tube. Director and tube are then passed through the cervix; the director is then steadied, and the tube pushed further on with forceps. The director is then inserted into the next hole in the tube, passed again through the cervix, and the tube again pushed off it. Thus the tube passes through the cervix as if it were solid, yet is allowed the freest excursion once it lies between the uterus and membranes. If any vessel, as in the placental site, is broken, the blood at once runs through the tube. The softness of the latter precludes the possibility of rupture of the uterus, and the membranes also are protected from injury. The method is highly efficient in bringing on labour.

### Estimation of the Date of Labour.

A new rule for the forecasting of the probable date of labour is given by Dr. E. MacDonald in the *American Journal of the Medical Sciences.* After the sixth month the rule is said to be extraordinarily exact, and has proved of great value in asylum practice for women who cannot give any connected account of their menstrual history. The rule is that the duration of pregnancy in lunar months is equal to the height of the uterus in centimetres, divided by 3.5. The uterus is said to grow fairly regularly 3.5 cm. per lunar month. The measurement is taken with the patient lying flat. One end of the tape is placed at the upper border of the symphysis, and the tape follows the contour of the uterus, save at the upper pole; here it is held out so as to touch the fingers, which sink vertically over the fundus and are set tangentially to its highest point. Multiparæ with lax abdominal walls and thin uteri should be supported at the side, so as to bring the occipitococcygeal axis of the pelvis into the long axis of the mother's body. As an illustration, if the fundus measures 26 cm. from the symphysis, the duration of pregnancy is 26 ÷ 3.5 or $7\frac{3}{7}$ lunar months. The patient has, therefore, $2\frac{4}{7}$ lunar months to go to term, or ten weeks and two days. When the measurement is at or near 35 cm., Dr. MacDonald never hesitates to induce labour if necessary, as the fœtus is then at full term.

### The Action of Apocynum.

Drs. Dale and Laidlaw have investigated the action of this drug, and their account of it is published in *Heart.* The active principle recently isolated from *Apocynum cannabinum* by Finnemore exhibits properties identical with those of the substance obtained from *Apocynum androsæmifolium* by Moore. These are named respectively Cynotoxin and Apocyamarin. Crystalline apocynin has but the feeblest physiological action when pure. The diuretic action of apocynum has earned for it in America the name of the "vegetable trocar." The action is in all respects a characteristic digitalis effect. The authors have not yet determined accurately the exact position of the drug in the digitalis series, but they find its vaso-constrictor action is considerably more powerful than that of strophanthin; its action on the heart is slightly weaker than that of the latter. On the other hand, it is excreted or destroyed with comparative rapidity, and there is experimental basis for the statement that apocynum is not cumulative in its action. The question whether it is excreted or destroyed within the body remains open for the present. It is thought that the employment of the pure active principle should eliminate the drawbacks which have hitherto restricted the use of this drug, for these are probably due to the presence of other constituents of an irritant nature in the crude extracts. The rapidity with which cynotoxin acts enjoins caution with respect to subcutaneous dosage: it is not suggested what the latter should be.

### Nickel Poisoning from Barley Water.

The increasing favour which barley water is receiving as a summer drink is one of those signs of the times which should make temperance advocates rejoice. This wholly admirable drink is often flavoured with the juice of limes or lemons, or with manufactured lime-juice. An experience contributed to the *Journal of the Royal Army Medical Corps* is useful as showing possibilities of the combination which, unless avoided, may conceivably create prejudice against barley water as a beverage. In the mess of a cavalry regiment stationed in India some barley water flavoured with the juice of fresh limes was placed in a large metal urn having a central chamber for ice; the urn was made of white metal coated with nickel. Within a quarter of an hour of partaking of this drink four officers felt very sick, and in one case the symptoms persisted until the following day. The same evening the mess sergeant drank half a pint, which had been standing in the urn all day, and was sick in five minutes. On analysis the barley water was found to contain nickel, no doubt dissolved by the citric acid of the lime-juice from the plating of the urn. Yet another danger of the same sort has recently been recognised in India, where tablets of acid sodium sulphate have been distributed for water-sterilising purposes. Water thus treated and contained in metal water-bottles may corrode the latter owing to the formation of sulphuric acid in it. Aluminium, zinc, and iron have been found thus dissolved in the water from bottles of those metals respectively.

